# The Preparation and Properties of Amino-Carboxymethyl Chitosan-Based Antibacterial Hydrogel Loaded with ε-Polylysine

**DOI:** 10.3390/foods12203807

**Published:** 2023-10-17

**Authors:** Yixi Li, Yulong Qiu, Hongman Hou, Gongliang Zhang, Hongshun Hao, Jingran Bi

**Affiliations:** 1 School of Food Science and Technology, Dalian Polytechnic University, No. 1, Qinggongyuan, Ganjingzi District, Dalian 116034, China; lyxixi3@163.com (Y.L.); qiuyulong2022@163.com (Y.Q.); zgl_mp@163.com (G.Z.); houhongman@dlpu.edu.cn (H.H.); beike1952@163.com (H.H.); 2 Liaoning Key Lab for Aquatic Processing Quality and Safety, No. 1, Qinggongyuan, Ganjingzi District, Dalian 116034, China

**Keywords:** hydrogel, amino-carboxymethyl chitosan, dialdehyde starch, antimicrobial peptide, bacterial inhibition

## Abstract

In this paper, amino-carboxymethyl chitosan (ACC) was prepared through amino carboxymethylation, which introduces -COOH and -NH_2_ groups to the chitosan (CS) chains. Meanwhile, dialdehyde starch (DAS) was produced by oxidizing corn starch using sodium periodate. To attain the optimal loading and long-time release of ε-polylysine (ε-PL), the ACC/DAS hydrogels were synthesized through the Schiff base reaction between the amino group on ACC and the aldehyde group in DAS. The molecular structure, microcosmic properties, loading capacity, and bacteriostatic properties of the four types of hydrogels containing different mass concentrations of ACC were investigated. The results showed that the dynamic imine bond C=N existed in the ACC/DAS hydrogels, which proved that the hydrogels were formed by the cross-linking of the Schiff base reaction. With the increasing mass concentration of the ACC, the cross-sectional morphology of the hydrogel became smoother, the thermal stability increased, and the swelling behavior was gradually enhanced. The tight network structure improved the ε-PL loading efficiency, with the highest value of 99.2%. Moreover, the loading of ε-PL gave the hydrogel good antibacterial properties. These results indicate that ACC/DAS hydrogel is potential in food preservation.

## 1. Introduction

Microbiologically derived natural bioactive ingredients have become popular in recent years for use in functional foods and food preservation due to their nutritional value and bioactivity. They are being used to meet people’s desire for a healthy lifestyle [1]. However, the application of these bioactive compounds in the food industry is hampered by their unstable nature, which results in poor solubility in water, high susceptibility to enzymes, and low thermostability [2]. ε-polylysine (ε-PL) was initially obtained from the *Streptomyces albulus* sp. *Lysinopolymerus* by the researchers Shima and Sakai [3]. It is classified as a cationic homo-polyamide and is composed of approximately 25–30 lysine residues, which are connected by an amide linkage that bridges the 3-amino and carboxyl groups [4]. The potent antimicrobial activity, low toxicity, and high water solubility of ε-PL make it an ideal option for preventing or controlling bacterial growth in food products [5]. However, the potent antimicrobial activity of the cationic ε-PL can be reduced in alkaline foods through the precipitation of the resulting electrostatic complexes, which prevents ε-PL from interacting with microbial surfaces [6].

Hydrogels are three-dimensional networks composed of hydrophilic polymer chains that are interconnected through physical or chemical connections [7]. The distinctive physical characteristics exhibited by hydrogels render them advantageous as vehicles for pharmaceuticals and physiologically active compounds. The porosity of hydrogel can be effectively controlled by manipulating the degree of cross-linking. This mechanism enables molecules of various sizes to penetrate the hydrogel structure. The diffusion, swelling control, and chemical processes such as hydrolysis or enzymatic hydrogel networks can alter the bioactive release rate from a hydrogel [8]. The Schiff base reaction, characterized by its rapid and mild nature, has found extensive utilization in hydrogel applications due to its favorable attributes. Chitosan (CS) is hydrophobic, biodegradable, biocompatible, non-toxic, and antibacterial, and the rich amino groups are widely used in Schiff base reactions [9]. Guan et al. introduced glutaraldehyde to prepare chitosan sodium alginate polyelectrolyte hydrogels via the Schiff base reaction, which compounded a sparse, porous, and amorphous structure, thereby improving their swelling behavior [10]. However, it is important to note that natural CS demonstrates limited water solubility and relatively low antibacterial efficacy. The enhancement of CS’s solubility and antibacterial efficacy can be achieved by synthesizing functional chitosan derivatives through chemical modification [11]. Amino-carboxymethyl chitosan (ACC) has superior biological and physicochemical characteristics compared to CS, thus making it a suitable option for numerous applications. By introducing more amino groups into the CS structure, the Schiff base reaction with aldehyde group compounds may be considerably enhanced while preserving the properties of the CS chain. Additionally, ACC has better biocompatibility, water retention capability, and greater viscosity than CS, thereby qualifying it to be used as a hydrogel matrix [12]. Dialdehyde starch (DAS) has excellent physical, chemical, and biochemical properties and is a starch modifier with a large number of reactive aldehyde groups. Luo et al. demonstrated that chitosan-dialdehyde starch membranes based on Schiff bases are biocompatible with common mammalian cells [13].

In order to fabricate a long-term release carrier for ε-PL, a bio-based hydrogel was constructed in this paper. Firstly, CS was modified through amino-carboxymethylation to increase the amino content of the polymer chain. Meanwhile, DAS was obtained from maize starch through the oxidation of NaIO_4_. The amino group located on ACC underwent a reaction with the aldehyde groups presented on DAS, resulting in the formation of a hydrogel through a Schiff base reaction. The characterization of ACC/DAS hydrogels with varying ACC concentrations was conducted using several analytical techniques, including scanning electron microscopy (SEM), Fourier transform infrared spectroscopy (FTIR), X-ray diffraction (XRD), and thermogravimetry (TGA), and their swelling characteristics were also analyzed. The loading capacity of ACC/DAS with ε-PL was quantified, and the antibacterial characteristics of the ACC/DAS/ε-PL hydrogels were examined. The aim of this study was to explore the potential applications of ACC based hydrogels in food preservation.

## 2. Materials and Methods

### 2.1. Materials

CS (80~97% degree of deacetylation and molecular weight = 30,000 Da) was obtained from Sangon Biotech (Shanghai, China). Corn starch was obtained from Yuanye Biotechnology (Shanghai, China). Sodium periodate (NaIO_4_, 98%) and ε-PL were obtained from Aladdin Biochemical Technology (Shanghai, China). Ethyl-3-(3-dimethylaminopropyl) carbodiimide hydrochloride (EDC, 98.5%) was obtained from MacLean Biochemical Technology (Shanghai, China). The chemicals ethylenediamine anhydrous (ED, 99%), chloroacetic acid, and dimethyl sulfoxide (DMSO, 99.5%) were obtained from the Damao Chemical Reagent Factory (Tianjin, China). *Staphylococcus aureus* (*S. aureus*, ATCC 6538) and *Escherichia coli* (*E. coli*, CICC 10670) were obtained from the China General Microbial Strain Repository Management Centre (Wuhan, China).

### 2.2. Synthesis of ACC and DAS

A solution of CS (5 g) was prepared by dissolving it in 50 mL of dimethyl sulfoxide (DMSO) at 30 °C for 60 min. Alkalized CS was prepared by adding sodium hydroxide (90% *w*/*v*) and mixing at 30 °C for 2 h. After the addition of 25 g chloroacetic acid, the mixture was stirred at 60 °C for 4 h, and the mixture was then filtered and rinsed with 75% volume ethanol. The purified material was subjected to a drying process at 50 °C for 12 h. Then, the solution underwent dissolution, followed by pH adjustment to 3.65 with the addition of hydrochloric acid with a concentration of 1 mol/L. This process resulted in the formation of a white precipitate. The precipitate was determined to be carboxymethyl chitosan (CC). The sample underwent two rinses using anhydrous ethanol, followed by a roasting process at 60 °C for 6 h. Following this, CC was dissolved in phosphate buffered saline (PBS) at a concentration of 2.5 wt% and a pH of 7.4. The solution was then agitated continuously for the duration of the night. ED was added at a molar ratio of 20:1 (ED:CC). Hydrochloric acid (HCl) was added to adjust the pH of the solution to 5.0. The compound EDC was subsequently introduced to the compound CC at a molar ratio of 2:1. After subjecting the solution to agitation at 37 °C for 4 h, it was thereafter subjected to dialysis for a period of 72 h in water. A dialysis membrane with a molecular weight cut off (MWCO) of 3.5 kDa was used to dialyze the solution. This continued until the dialysate no longer included any traces of ethylenediamine and EDC. The acquisition of ACC was achieved through the process of freezing the reaction product at a temperature of −80 °C.

The corn starch (2 g) was dissolved in 23 mL of hydrochloric acid solution with a final concentration of 0.6 mol/L. NaIO_4_ was then added at a molar ratio of 2:1 (NaIO_4_:starch) and the mixture was stirred at 40 °C for 2 h. Next, the resultant product underwent three washes with deionized water and was subjected to drying at 40 °C for a period of 12 h. The resulting product was identified as DAS.

### 2.3. Determination of Amino Content of ACC and Aldehyde Group Content of DAS

Initially, varying concentrations (1 mL) of β-alanine solution were combined with 1 mL of sodium bicarbonate (4 wt%) and 1 mL of 2,4,6-trinitrobenzenesulfonic acid (0.1 wt%). The mixture was then reacted at 40 °C for 2 h before being diluted fivefold. The absorbance at 415 nm was measured using a UV-vis spectrophotometer (UV-1750, Shimadzu, Japan), and a standard curve was subsequently developed with the following formula: y = 1.3111x + 0.2936 (R^2^ = 0.9991), where y is the absorbance at 415 nm and x is the molar concentration of β-alanine. To determine the amino content of ACC, the ACC was substituted for β-alanine.

The aldehyde group content of DAS was determined using the alkali solubilization method by dissolving 0.2 g of DAS in 10 mL NaOH (0.25 mol/L), which was heated at 70 °C for 2 h and then cooled. Next, 15 mL of H_2_SO_4_ (0.125 mol/L) was added to the solution and shaken well until turning light-yellow. Then, 0.2 g of activated carbon was added and the solution was shaken for 2 min. The solution was filtered and five drops of phenolphthalein were added and titrated with 0.1 mol/L NaOH. The aldehyde group content was calculated using the Formula (1):

(1) $$-CHO(\%)=\frac{\left(C_{1}V_{1}+C_{2}V_{2}-2C_{3}V_{3}\right)}{m\times 1000}\times 100$$

*C*_1_ is the concentration of NaOH when dissolving DAS (0.25 mol/L), *V*_1_ is the volume of NaOH (10 mL), *C*_2_ is the concentration of NaOH used in the titration (0.1 mol/L), *V*_2_ is the volume of NaOH solution during the titration, *C*_3_ is the concentration of H_2_SO_4_ (0.125 mol/L), *V*_3_ is the volume of H_2_SO_4_ (15 mL), *m* is the mass of DAS, and 160 is the average relative molecular mass of DAS glucose unit.

### 2.4. Preparation of ACC/DAS Hydrogels

Solutions of ACC with concentrations of 4.5 *w*/*v*%, 6 *w*/*v*%, 7.5 *w*/*v*%, and 9 *w*/*v*% were prepared by dissolving 4.5 g, 6 g, 7.5 g, and 9 g of ACC in 100 mL of PBS at a pH of 7.4, respectively. The DAS solution with a concentration of 6 wt% was prepared by dissolving 6 g of DAS in 100 mL of PBS. Next, 10 mL ACC solutions were placed in an acrylic plate with a 90 mm diameter and dried at 40 °C for 30 min until the ACC solutions were semi-solidified. Then, 10 mL of DAS solution was poured on top of the ACC layer and dried at 40 °C for 3 h to obtain four hydrogels with molar ratios of 1:1.6, 1:1.25, 1:1, and 1:0.6 of -NH_2_:-CHO, named as the ACC_1_/DAS, ACC_2_/DAS, ACC_3_/DAS, and ACC_4_/DAS hydrogels.

### 2.5. Characterizations of ACC, DAS, and ACC/DAS Hydrogels

The ACC/DAS hydrogels were placed in liquid nitrogen and rapidly frozen before being sectioned. Scanning electron microscopy (SEM, JSM-7800F, JEOL, Tokyo, Japan) was used to assess the morphological features of the ACC/DAS hydrogels. The FTIR spectra of the ACC, DAS, and ACC/DAS hydrogels were obtained from 4000 to 400 cm^−1^ with a range resolution of 4 cm^−1^ using an FTIR spectrometer (Spectra II, PerkinElmer, Waltham, MA, USA). The XRD profiles of the ACC, DAS, and ACC/DAS hydrogels were collected at ambient temperature via a diffractometer (XRD-6100, Shimadzu, Kyoto, Japan) with a scanning range of 5° to 60° at a rate of about 5° per minute. The thermal stability of the ACC/DAS hydrogels was measured using a thermogravimetric analyzer (TGA550, TA, Newcastle, DE, USA) with a temperature range of 0–600 °C, a heating rate of 30 °C/min, and a nitrogen flow rate of 20 mL/min. For the determination of the mechanical properties, the hydrogels were cut into strips of 10 × 100 mm. The mechanical properties of the ACC/DAS hydrogels were tested using an automated tensile testing machine (INSTRON5965, Instron, Norwood, MA, USA), with an initial distance of 40 mm between the clamping plates and at a testing speed of 50 mm/s. The test was carried out at a speed of 1.5 m/s.

### 2.6. Swelling Degree of ACC/DAS Hydrogels

The ACC_1_/DAS, ACC_2_/DAS, ACC_3_/DAS, and ACC_4_/DAS hydrogels were immersed in deionized water for 10, 20, 30, 40, 50, 60, 70, 80, 90, 100, 110, and 120 min, respectively. Then, the hydrogels were taken out from the solution and promptly weighed through the elimination of surface moisture using filter paper [14]. The degree of swelling (SW) was determined using the following Formula (2):

(2) $$Swell\;degree(\%)=\frac{W_{S}-W_{D}}{W_{D}}\times 100$$

*W_S_* is the weight of the hydrogel subsequent to the process of swelling and *W_D_* is the weight of the hydrogel in its dry state prior to swelling.

### 2.7. Loading Efficiency of ACC/DAS Hydrogels

The ε-PL solution (2 mg/mL) was diluted with 1 mol/L PBS (pH 6.9) to yield ε-PL standard solutions with concentrations of 0.02, 0.05, 0.08, 0.1, 0.15, 0.2, and 0.25 mg/mL, respectively. Then, 0.2 mL of each standard solution was added to 0.8 mL of 0.5 mmol/L methyl orange. The samples were shaken for 30 min at 30 °C; they were centrifuged at 8000 rpm for 5 min, the supernatant was diluted 10 times and the absorbance was read at A_1_ at 465 nm. The absorbance A_0_ was measured using a mixture of PBS and methyl orange as the control, and the UV spectrophotometer was zeroed with PBS. The standard curve—represented by the equation y = 1.8277x + 0.0368 (R^2^ = 0.9938)—was derived using the mass concentration of ε-PL as the independent variable and the difference in absorbance ΔA = (A_0_ − A_1_) as the dependent variable.

ACC/DAS/ε-PL hydrogels are made by dissolving 0.2 g of ε-PL in 10 mL of the prepared ACC solution, as described in 2.4. Four ACC/DAS hydrogels with different ACC contents were added to a centrifuge tube containing 10 mL of deionized water, respectively. After shaking for 60 s, the samples underwent centrifugation at a speed of 10,000 revolutions per minute for a duration of 3 min [15]. The mass concentration of ε-PL in the supernatant was determined by means of colorimetric analysis with methyl orange. A volume of 0.2 mL of the supernatant was combined with 0.8 mL of the methyl orange solution with a concentration of 0.5 mmol/L. The supernatant underwent dilution 10 times, and the absorbance at a wavelength of 465 nm was quantified using a UV spectrophotometer. The absorbance value at OD_465_ was added into the ε-PL standard curve to obtain the ε-PL concentration; the loading efficiency was computed using the following Formula (3):

(3) $$Loading\;effciency\left(\%\right)=\frac{M_{0}-C_{s}V}{M_{0}}\times 100$$

where the symbol *M*_0_ is used to represent the initial mass of ε-PL added. *C_s_* is the weight concentration of ε-PL in the supernatant, and *V* is the volume of the supernatant (10 mL).

### 2.8. Antibacterial Activity of ACC/DAS Hydrogels

*Staphylococcus aureus* and *Escherichia coli* were selected as the experimental bacteria. They were cultured in liquid LB medium until logarithmic growth. The ACC/DAS/ε-PL hydrogels were cut into 2 × 3.5 cm rectangles and added into bacterial culture medium. The samples were subjected to incubation at a temperature of 37 °C, while being agitated at a speed of 50 revolutions per minute. A volume of 0.2 mL of the culture was extracted at time intervals of 6, 8, 10, 12, 24, 48, and 60 h, and ACC/DAS hydrogels without ε-PL addition were used as the control. The absorbance at a wavelength of 600 nm was subsequently determined using a UV-visible spectrophotometer [16]. Formula (4) for calculating the antimicrobial rate is as follows:

(4) $$Antibacterial\;rate\;(\%)=\frac{{OD}_{1}-{OD}_{2}}{{OD}_{1}}\times 100$$

In the equation, *OD*_1_ represents the bacterial optical density seen in the control group, whereas *OD*_2_ represents the bacterial optical density seen in the group treated with the hydrogels.

### 2.9. Statistical Analysis

All the experiments were performed in at least three independent trials. The statistical analyses were conducted using SPSS 16.0. (SPSS Inc., Chicago, IL, USA).

## 3. Results

### 3.1. Characterizations of ACC and DAS

**Figure 1:**
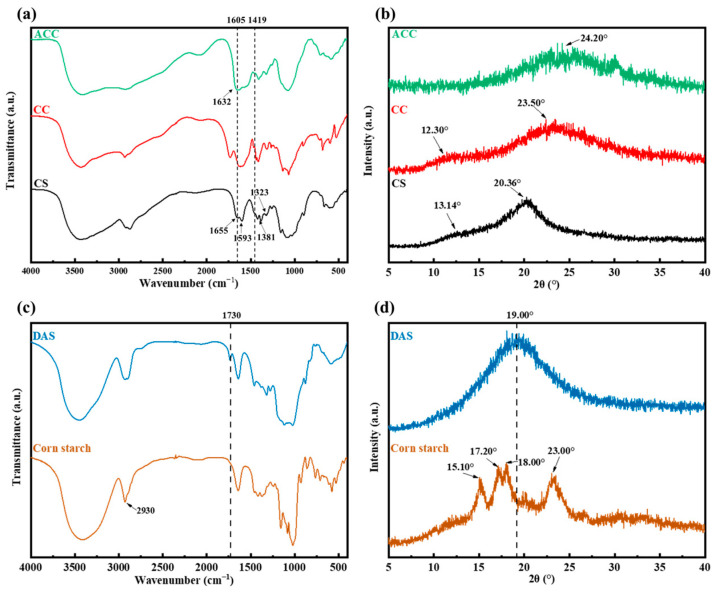
(**a**) FTIR spectra; (**b**) XRD patterns of CS, CC, and ACC; (**c**) FTIR spectra; (**d**) XRD patterns of corn starch and DAS.

ACC is a class of amination derivatives following the carboxymethylation of CS, containing -COOH and -NH_2_ functional groups. Through the determination using trinitrobenzene sulfonic acid, the amino content of ACC was 0.83 ± 0.02 mmol/g. In Figure 1a, the FTIR spectra of CS, CC, and ACC reveal distinct absorption bands at 1655, 1593, 1323, and 1381 cm^−1^, which are attributed to amides I, II, III, and the -CH_3_ vibration band of the CS chain, respectively. Both the CC and ACC spectra exhibited two prominent peaks at 1605 cm^−1^ and 1419 cm^−1^. The results are consistent with the description of Fan et al. [17]. These peaks can be attributed to the asymmetrical and symmetrical stretching of the -COO groups [18]. The observed absorption peak at 1632 cm^−1^ in the ACC spectra indicated the stretching vibration associated with the amide group. This finding suggests that the grafting of ethylenediamine onto CC has been accomplished effectively. It is evident that ACC has a notably greater amino content in comparison to CS [19]. Figure 1b displays the X-ray diffractograms of CS, CC, and ACC. There were noticeable differences in the peak height, size, and position between the CS and ACC. The CS sample displayed two prominent peaks at 2θ = 13.1° and 23.50°, indicating its crystalline nature [20]. On the other hand, the CC sample revealed two distinct peaks at 2θ = 12.30° and 20.36°. These peaks can be explained by the abundance of -OH and -NH_2_ groups within the CS molecule, which facilitate the creation of many hydrogen bonds [21]. The incorporation of carboxymethyl and amino groups into the structural unit of ACC resulted in a weakening of the hydrogen bonding interactions between the hydroxyl groups present on the chitosan chain. As a consequence, there was a decrease in the degree of crystallinity observed in chitosan, as evidenced by the broadening of the diffraction peaks for ACC [22]. In addition, the crystallinity of ACC was lower than that of CC due to the introduction of amino groups.

DAS is a significant type of modified starch that is produced by oxidizing starch with the potent oxidizing agent periodate. Using the alkali solubilization method, the aldehyde group contents of DAS were determined as 89.8 ± 0.77%. Analysis of the structural modifications of starch subjected to oxidation through sodium periodate was conducted using FTIR, as depicted in Figure 1c. The peak at around 3310 cm^−1^ was associated with the vibrational stretching of -OH, while the peak at 2930 cm^−1^ was assigned to the symmetric and asymmetric stretching vibrations of the C-H group. In contrast to native starch, the FTIR analysis of DAS revealed a significant peak at 1730 cm^−1^, which was related to the stretching vibration of C=O [23]. This would suggest that the oxidation of starchy maize by NaIO_4_ was effective [24]. The composition of the starch granules included both crystalline and non-crystalline regions. The starch has a crystalline structure of type A, as evidenced by the presence of XRD peaks at approximately 15.10°, 17.20°, 18.00°, and 23.00° (Figure 1d) [25]. The oxidation and acidic hydration events of corn starch result in the conversion of the hydroxyl group to an aldehyde. This chemical transformation causes the hydrogen bond between the starch components to break down, ultimately disrupting the crystal structure. The disappearance of the diffraction peaks intrinsic to starch in the XRD pattern of DAS after acidolysis and oxidation provides evidence that the A-type crystallization of the starch was disturbed by sodium periodate [26].

### 3.2. Preparation and Characterizations of ACC/DAS Hydrogels

**Scheme 1:**
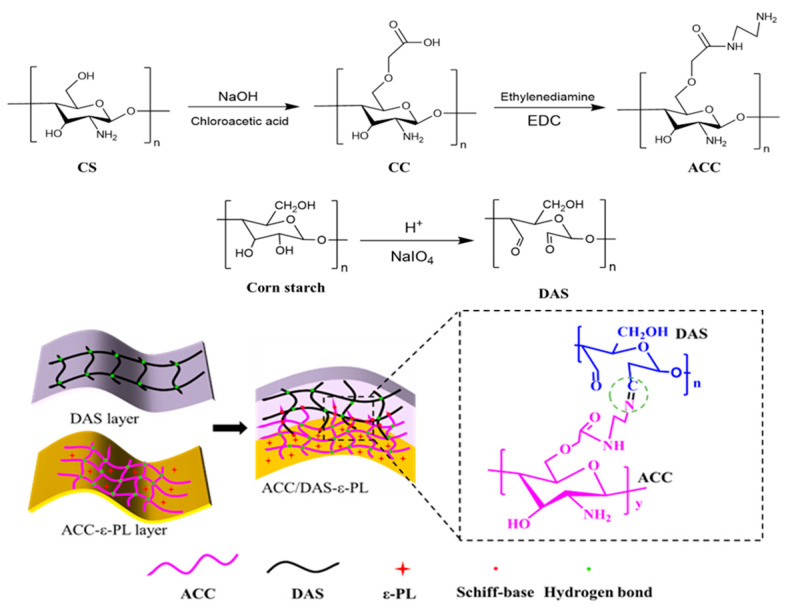
Preparation approach of ACC/DAS hydrogels.

As depicted in Scheme 1, the CS surface exhibits a substantial presence of reactive groups—namely amino and carboxyl groups—which therefore undergo modifications through chemical reactions. In this thesis, a two-step process of carboxymethylation and amino-carboxymethylation was used to enhance the amino group on the surface of chitosan. Simultaneously, the hydroxyl groups within the corn starch molecules were aldehydized through the process of sodium periodate oxidation. Afterwards, the amino group on the ACC surface was cross-linked with the aldehyde group on the DAS to form imine bonds, and a three-dimensional network skeleton structure of long-chain polysaccharides was constructed. The ε-PL was efficiently immobilized on the hydrogel matrix through hydrogen bonding, thus preparing an ACC/DAS/ε-PL system with long-lasting bacterial inhibition, in order to build a hydrogel that can effectively load and continuously deliver natural active substances.

**Figure 2:**
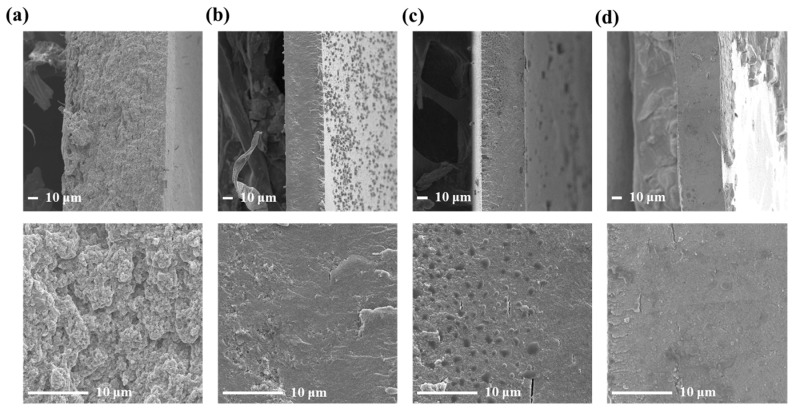
SEM image of (**a**) ACC_1_/DAS; (**b**) ACC_2_/DAS; (**c**) ACC_3_/DAS; (**d**) ACC_4_/DAS hydrogels.

In Figure 2, the cross-section morphologies of the four ACC/DAS hydrogels were exhibited. When the molar ratio of the amino group on the ACC molecule to the aldehyde group on the DAS surface is 1:1.6, the cross-section of the hydrogel ACC_1_/DAS is very rough and there is a large amount of visible aggregation. As the ACC content increases, the aggregation on the cross-section of the ACC_2_/DAS hydrogel decreases and becomes more lamellar and denser. When the molar ratio of -NH_2_ to -CHO reached 1:0.6, the cross-sectional microstructure of ACC_4_/DAS was flat and smooth, with no aggregation. This may be due to the increased Schiff base reaction enhanced cross-link density, which built a tight and dense 3D network structure.

**Figure 3:**
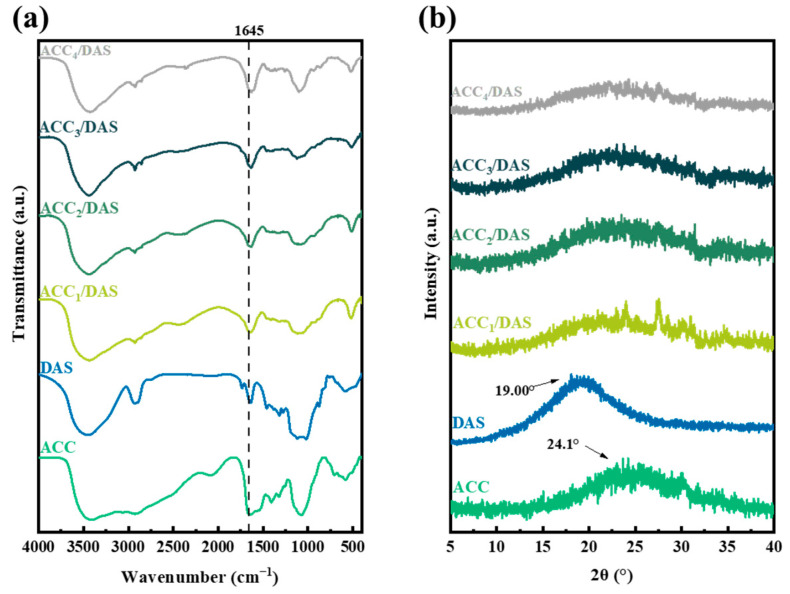
(**a**) FTIR spectra; (**b**) XRD patterns of ACC/DAS hydrogels.

In the FTIR spectrograms of the four ACC/DAS hydrogels with increasing amino contents in ACC (Figure 3a), the 1735 cm^−1^ absorption band of the aldehyde stretching vibration and the 1545 cm^−1^ absorption band of the amino stretching vibration gradually weakened due to the Schiff base reaction, while the 1630 cm^−1^ absorption band belonging to the imine group was correspondingly enhanced. This indicates that imine Schiff base crosslinks (-NCH-) are established between the ACC and DAS within the ACC/DAS hydrogel network [27].

The XRD patterns of the ACC/DAS hydrogels are shown in Figure 3b. When the ACC reacted with the DAS to form the hydrogel, the peaks of DAS at 19.0° and ACC at 24.1° disappeared. The XRD pattern of the ACC/DAS composite exhibits broad signals, indicating its amorphous structure. These results indicated that a strong Schiff base interaction between the DAS and ACC molecules occurred, and the long chains of the two molecules were arranged both separately and entangled with each other, thus disrupting the original arrangement [28].

**Figure 4:**
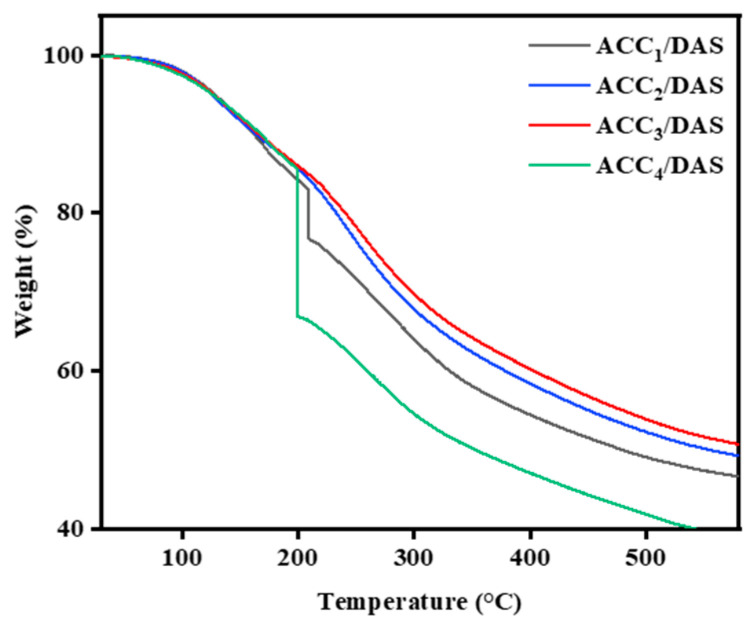
The thermogravimetric analysis of ACC/DAS hydrogels.

The TGA plot in Figure 4 depicts the thermal decomposition pattern of the ACC/DAS hydrogels. There are two stages of mass loss for ACC/DAS hydrogels containing different concentrations of ACC. Between 80 and 160 °C, the hydrogel experienced weight loss caused by the evaporation of residual water molecules. This weight loss indicated a significant hydrogen bonding pattern in the hydrogel [29]. The second phase of degradation occurs rapidly between temperatures of 200 to 500 °C. In addition, the hydrogel undergoes a major mass loss phase, with the maximum weight loss rate at 203 °C [30]. At this point, it is believed that the thermal decomposition of the macromolecular backbone in the hydrogel substrate occurs, mainly due to the degradation of the glucosamine and acetylated groups [31]. Additionally, it is evident that the thermal stability of the hydrogel demonstrated a notable enhancement when the molar ratio of -NH_2_:-CHO rose from 1:1.6 to 1:0.6. Conversely, a substantial decline in the thermal stability of the hydrogel is observed when the molar ratio of -NH_2_:-CHO is 1:0.6. These results indicated that a sufficient amount of -NH_2_ probably leads to a strong Schiff base reaction between the polymer components in the hydrogel, which builds a dense structure of the hydrogel and possesses thermal stability [32]. However, the strongest Schiff base reaction occurred in the ACC_4_/DAS hydrogel, which produced the superfluous Schiff base fractures in the second phase and a decrease in the thermal stability [33].

### 3.3. Swelling Behavior of the Hydrogels

**Figure 5:**
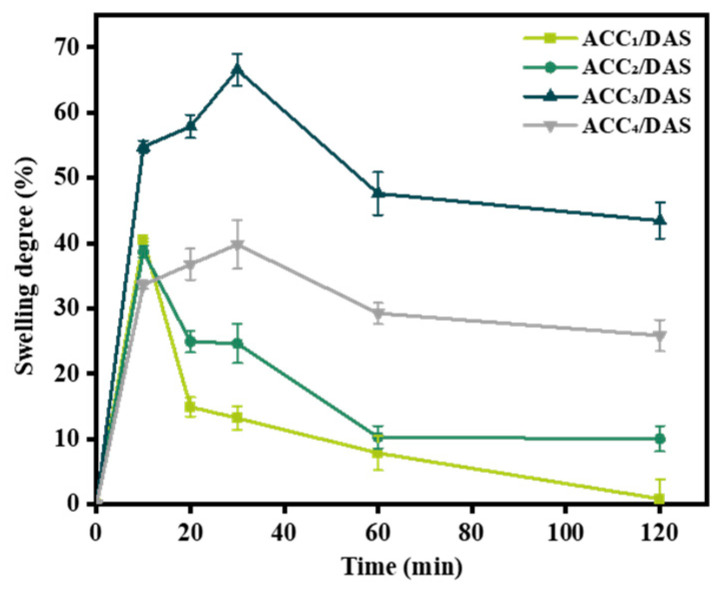
Degree of swelling plotted against time for ACC/DAS hydrogels.

The appearance of swelling in hydrogels is widely recognized as a crucial characteristic of hydrogels, and the hydrophilic nature of ACC/DAS hydrogels allows small molecules such as water to enter the three-dimensional network structure through swelling, providing a scaffolding structure for the loading of active substances. As shown in Figure 5, all four hydrogels swelled rapidly within 10 min, with the highest swelling degree of 67% for the ACC_3_/DAS hydrogels. A decreasing trend in the swelling curve of ACC_1_/DAS and ACC_2_/DAS was clearly observed between 10 and 30 min, while the swelling degrees of ACC_3_/DAS and ACC_4_/DAS were still increasing due to the higher -NH_2_ contents. When hydrogels are submerged in water, water molecules are forced to move into the polymer structure due to the presence of an osmolality gradient. The phenomenon of water diffusion leads to the expansion of hydrogels, hence causing an increase in the pore size within the hydrogel. ACC_3_/DAS exhibited a longer swelling equilibrium time than the other hydrogels. Meanwhile, it is clear that the level of expansion of the hydrogel increases when the molar ratio of -NH_2_:-CHO in the hydrogel is 1:1.6, 1:1.25, and 1:1, while the swelling degree of the ACC_4_/DAS hydrogel is the lowest when the molar ratio of -NH_2_:-CHO in the hydrogel is 1:0.6. This was due to the excessive crosslinking of the Schiff bases, which resulted in the polymer chains’ tight cross-linked network, leading to a reduced pore size and poor flexibility, which in turn restricts the passage of water molecules, and the swelling properties of the hydrogels are limited by the spatial site resistance effect [34].

The mechanical properties of the four ACC/DAS hydrogels with different ACC contents are shown in the Table 1, and it can be seen that the tensile strength and elastic modulus of the hydrogels improved significantly, while the breaking elongation gradually reduced with the increase in the amino acid content in the ACC, which may be due to the formation of the Schiff base bond through the reaction of -NH_2_ and -CHO in the hydrogel. These results indicated that the Schiff base reaction could contribute to the cross-link of the network, leading to the toughness of the ACC/DAS hydrogel being enhanced.

### 3.4. Loading Efficiency of the Hydrogels

**Figure 6:**
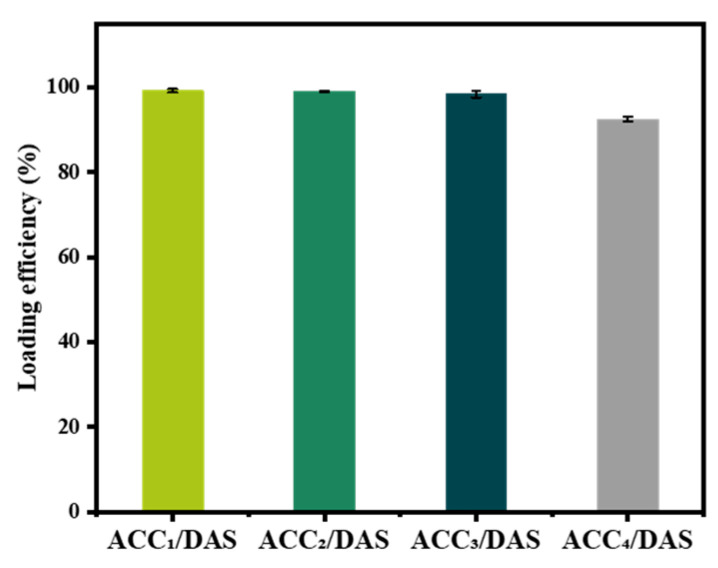
The ε-PL loading efficiency of ACC/DAS hydrogels.

As a typical natural cationic peptide with excellent antibacterial activity, ε-PL can be adsorbed onto the surface of cell membranes, thereby disrupting the cell membrane structure, inhibiting enzyme activity, interfering with normal gene expression, and ultimately destroying microorganisms. The ACC/DAS hydrogels were used as carriers of ε-PL. With molar ratios of -NH_2_:-CHO of 1:1, the loading efficiency of the ε-PL in the hydrogels reached up to 99.2% (Figure 6). The Schiff base cross-linked structure exhibits a robust inhibitory effect on the diffusion of ε-PL by means of steric hindrance. However, the ACC_4_/DAS hydrogel with a -NH_2_:-CHO molar ratio of 1:0.6 resulted in a significantly lower loading efficiency. The reduced physical encapsulation rate of the ε-PL in the ACC_4_/DAS hydrogel may be attributed to the excessive crosslinks between the polymer chains within the hydrogel.

### 3.5. Antibacterial Activity of the Hydrogels

**Figure 7:**
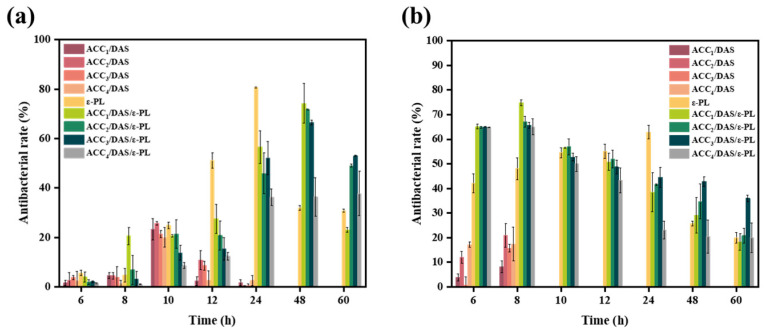
Antibacterial rate of ACC/DAS/ε-PL hydrogels against (**a**) *S. aureus* and (**b**) *E. coli* for 60 h.

Hydrogels possess notable appeal as carriers of active compounds owing to their considerable surface area to volume ratio and capacity for structural manipulation [35]. The ACC/DAS hydrogels without ε-PL also exhibited antibacterial properties due to the amino group on the CS chain; however, the antibacterial properties were only temporary: for 24 h against *S. aureus* and 8 h against *E. coli*. When the ACC/DAS/ε-PL hydrogel was added into the medium, water molecules diffused from the medium into the hydrogel network under the osmotic pressure gradient, and the release of ε-PL with antibacterial properties was caused by the swelling of the hydrogel. Figure 7a shows the bacteriostatic effect of the ε-PL and the four ACC/DAS hydrogels loaded with ε-PL against *Staphylococcus aureus*. Among them, the medium with only the addition of ε-PL reached the maximum inhibition rate of 80.67 ± 0.23% at 24 h; then, the inhibition rate gradually decreased. The inhibition rate was only 31.88 ± 0.88% at 48 h, which may be due to the depletion of ε-PL. The maximum inhibition rate of the ACC_2_/DAS/ε-PL and ACC_3_/DAS/ε-PL hydrogels was 71.84 ± 0.21% and 66.43 ± 0.92% at 48 h, respectively, and the antibacterial activities still maintained at 60 h, with the values of 49.03 ± 0.61% and 52.96 ± 0.20%, respectively, suggesting that good swelling behavior is beneficial for the long-time release. The ACC_4_/DAS hydrogel had the lowest inhibition rate, indicating that the immoderate crosslinked network was too tight and firm to allow the entry of water molecules, which limited the release of ε-PL. Figure 7b shows the inhibitory effect of the ACC/DAS hydrogels loaded with ε-PL on *E. coli*, and it was found that all of the inhibition rates of the hydrogels on *E. coli* reached the highest at 8 h, with the value of 74.81 ± 1.26%, and the antibacterial activities still maintained at 60 h, with the values of 36.02 ± 1.28%. The bacterial inhibition experiments show that the ACC/DAS/ε-PL hydrogels have a significant effect in inhibiting the growth of both *S. aureus* and *E. coli*. In addition, the hydrogel network formed by the Schiff base reaction is able to prolong the release of ε-PL and extend the time of its action, which has the potential to be used as active food packaging.

## 4. Conclusions

This study focused on preparing ACC/DAS hydrogels through a Schiff base reaction to attain the optimal loading efficiency and prolonged release of ε-PL. With the increase in the ACC mass concentration, the hydrogel structure became denser, with good thermal stability, and the swelling degree was also enhanced, with the highest value of 67%. The maximum ε-PL loading efficiency of the ACC/DAS hydrogels reached 99.2%. The good swelling behavior of the ACC/DAS hydrogels was beneficial for the long-time release of ε-PL, which prolonged the bacteriostatic time against both *S. aureus* and *E. coli*. However, the excessive Schiff base crosslink of the ACC/DAS hydrogel limited the loading and release of ε-PL. These results demonstrated that the ACC/DAS hydrogels were good candidates for use as a good carrier for active substance delivery and active food packaging for food preservation.

## Figures and Tables

**Table 1:** The mechanical properties of ACC/DAS hydrogels.

Samples	Tensile Strength (MPa)	Breaking Elongation (%)	Elastic Modulus (MPa)
ACC_1_/DAS	43.3 ± 7.4 ^a^	40.5 ± 0.03 ^a^	81.47 ± 5.4 ^a^
ACC_2_/DAS	43.7 ± 5.7 ^a^	10.2 ± 0.04 ^b^	586.2 ± 3.7 ^b^
ACC_3_/DAS	59.3 ± 7.6 ^b^	7.6 ± 0.04 ^b^	663.7 ± 1.9 ^b^
ACC_4_/DAS	63.2 ± 8.4 ^c^	6.4 ± 0.01 ^b^	741.1 ± 2.3 ^b^

Different letters in different columns represent significant differences between samples (*p* < 0.05).

## Data Availability

The data used to support the findings of this study can be made available by the corresponding author upon request.
